# Is managerial ability a catalyst for driving digital transformation in enterprises? An empirical analysis from internal and external pressure perspectives

**DOI:** 10.1371/journal.pone.0293454

**Published:** 2024-02-13

**Authors:** Weilin Wu, Jing Song, Lei Lu, Hongxia Guo

**Affiliations:** 1 Institute for China Common Prosperity Research, School of Economics, Jiaxing University, Jiaxing, Zhejiang, China; 2 School of Business, Macau University of Science and Technology, Avenida WaiLong, Macau, China; 3 School of Psychological and Cognitive Sciences, Beijing Key Laboratory of Behavior and Mental Health, Peking University, Beijing, China; University of Naples Federico II: Universita degli Studi di Napoli Federico II, ITALY

## Abstract

In a dynamic and competitive business environment, managerial ability emerges as a pivotal strategic factor for capitalizing on new opportunities within the technological revolution and digital transformation of enterprises. Based on data from Chinese A-share listed firms spanning from 2009 to 2019, this study integrates insights from the upper echelons theory and the behavioral theory of the firm to investigate the moderating roles of historical aspiration shortfalls and industrial competitiveness on the relationship between managerial ability and enterprise digital transformation from internal and external pressure perspectives. Our findings indicate a positive impact of managerial ability on digital transformation. The relationship between managerial ability and digital transformation is reinforced by historical aspiration shortfalls; nevertheless, industrial competitiveness has attenuated the aforementioned relationship. This study contributes to a better understanding of the strategic implications of managerial ability within the context of organizational innovation strategies. It offers valuable insights into the decision-making processes of firms as they navigate the challenges of digital transformation within an ever-evolving business environment.

## 1. Introduction

Digital transformation is an effective way to advance industrial upgrading and re-engineer the model of economic growth, as well as a significant role in facilitating the superior development of businesses [1, 2]. In the era of the digital economy, digital transformation has been taken as a tool to achieve competitive advantages over industries and countries [3, 4]. However, digital transformation is also a high-risk strategy [2], which poses new challenges for managers and reveals the importance of studying this new strategic transformation paradigm [5]. Digital transformation is related to transforming the industrial management model into a digital one, which requires enterprises to introduce digital technologies into current business processes, marketing channels, and supply chains [6, 7]. Prior literature explored these internal factors (e.g., organizational form, organizational culture, and digital technology) and external factors (e.g., national systems and industrial technology dynamics) influencing digital transformation from different fields and perspectives [1, 2, 8]. However, they ignored the effect of managers on digital transformation and failed to demonstrate the influencing path of managers [9].

Managers are the decision-makers and executors of corporate strategy decisions. Their capabilities are critical factors in assessing the performance of digital transformation. Recent studies suggest that managerial ability is a significant perspective in exploring how firms make strategic decisions, such as the digital transformation strategy [10, 11]. Managerial ability is defined as the capacity of the top management team to effectively convert corporate resources, encompassing capital, labor, and innovative assets, into revenue, profit, or firm value in comparison to industry peers [12–14]. The upper echelons theory proposes that managers make decisions based on their perception, which means organizational behaviors (i.e., digital transformation) can be a reflection of their abilities [15]. Previous research has focused on the impact of explicit managerial traits, with less attention paid to implicit managerial traits [16]. Implicit traits of managers, such as ability, quality, and reputation, are the key factors that shape their perceptions and behaviors [12, 17]. Therefore, it is a valuable study to identify the relationship between managerial ability and digital transformation, providing new evidence for further understanding the role of managerial ability in the digital transformation process [18].

In addition, this study delves into the boundary conditions of how managerial abilities influence digital transformation from internal and external pressure perspectives. On one hand, the behavioral theory of the firm posits that organizations manifest their objectives through the establishment of aspiration levels. These aspiration levels influence a firm’s assessment of its actual performance, and when actual performance falls short of these aspirations, decision-makers tend to initiate alterations in existing strategic practices. This, in turn, prompts the organization to engage in risky strategic change behaviors, ultimately driving the firm towards the implementation of digital transformation initiatives. On the other hand, the resource-based view suggests the sustainability of a firm’s profits is influenced by the interplay between the enterprise’s resources, capabilities, and the structure of the industry in which it operates [19]. The efficacy of organizational capabilities (e.g., managerial ability) in enhancing business management efficiency is contingent upon the competitive dynamics of the operational environment [20]. Therefore, this study scrutinizes their moderating influences on the relationship between managerial ability and digital transformation, with a particular focus on the interplay of historical aspiration shortfalls and industrial competitiveness from internal and external pressure perspectives.

This study exploits research data from Chinese A-share listed firms between 2009–2019 and obtains managerial ability and digital transformation information from these firms’ prospectuses, annual reports, and relevant databases for empirical research. Based on the upper echelons theory and the behavioral theory of the firm, we discuss the influence of managerial ability on digital transformation. In light of the potential impact stemming from the internal and external organizational environment upon this nexus, we further introduce historical aspiration shortfalls and considerations of industrial competitiveness as moderating variables. These variables serve as instruments to scrutinize the contextual conditions governing the relationship between managerial ability and digital transformation. In light of the potential impact stemming from the internal and external organizational environment upon this nexus, we further introduce historical aspiration shortfalls and considerations of industrial competitiveness as moderating variables. These variables serve as instruments to scrutinize the contextual conditions governing the relationship between managerial ability and digital transformation. Furthermore, our research endeavors encompass an exploration of the variations within the relationship between managerial competence and digital transformation within firms characterized by differing configurations of property rights and varying levels of corporate governance.

This study seeks to address a notable gap in the existing literature by enhancing our comprehension of the implications stemming from implicit traits of managers, such as managerial ability, upon organizational strategy. Additionally, there exists a dearth of comprehensive research frameworks investigating the combined influences of both internal and external organizational environments on the digital transformation process. Our study endeavors to address these knowledge gaps by posing questions concerning the distinctions that may arise between internal and external challenges encountered by organizations, and the roles these challenges assume within the digital transformation.

In answering these questions, this study makes some contributions. First, we build a direct link between managerial ability and digital transformation, and examine the conditions where managerial ability benefits firms, enriching the literature on the antecedents of digital transformation. Our study expands on the new impact of individual ability on organizational innovation strategies, such as the interactions between managerial abilities and digital transformation [21]. Second, drawing upon the behavioral theory of the firm, this study examines the moderating influences of internal and external pressure factors (i.e., historical aspiration shortfall and industrial competitiveness) on enterprise development, thereby refining the relationship between managerial ability and enterprise digital transformation. This study contributes to a more comprehensive comprehension of the impacts exerted by internal operational pressure and external industrial pressure upon the execution of organizational innovation strategies. Lastly, this study exploits the upper echelon theory and the behavioral theory of the firm in an integrated framework, increasing relevant contextual conditions for the upper echelon theory [22]. Our research contribution to the behavioral theory of the firm lies in the integration of both internal and external developmental scenarios within a comprehensive perspective that considers the firm’s internal and external pressures as a holistic entity. Our theoretical framework and its empirical test enable us to contribute to the theoretical integration of the upper echelon theory and the behavioral theory of the firm by exploring the effect of managerial ability on digital transformation from internal and external pressure perspectives. It responds to the call of existing literature that the theoretical explanation of digital transformation should be developed further [23].

## 2. Theory and hypotheses

### 2.1 Managerial ability and digital transformation

Managerial ability combines a manager’s knowledge, experience, and values, reflecting the manager’s ability to deal with complex issues and make decisions. The managerial ability has a vital role in strategic decision-making. Upper echelon theory suggests that a manager’s personal characteristics can have a significant impact on the development of the organization. Managerial ability, as a specific characteristic of managers, plays a crucial role in strategic decision-making [24]. Numerous studies have shown that managers with more vital managerial skills can implement better strategies [25, 26]. First, managers with higher abilities are motivated by more ideal incentives firms provide [27]. Increasing incentives is an effective way to mitigate agency problems and helps managers overcome risk-averse tendencies, because managerial incentive programs are based on tolerance for early failures and rewards for long-term success (e.g., stock options). Prior research shows that companies have incentive programs for high-ability managers to ensure managers are willing to make investment decisions with long-term value in the decision-making process to derive more direct benefits from future value appreciation [28, 29]. Therefore, the managerial capability will play a more active role through incentives, which will help to promote digital transformation.

Second, managers with higher abilities will enhance the entrepreneurial orientation of the company [30]. Prior research demonstrates that managers with more potent managerial skills are more willing to take risks and contribute to constructing an entrepreneurial orientation [31, 32]. For example, managers with higher abilities are active managers who make superior venture capital decisions in the investment industry. Chemmanur et al. found that top managers with a better reputation can select better projects [33]. Managers with an entrepreneurial strategy orientation are more optimistic about the outcomes and rewards of pursuing new opportunities [34, 35], and they are more likely to invest in digital transformation projects. Further, managers with an entrepreneurial strategy orientation are more tolerant of risk, which leads managers with high abilities to make risky investments [36, 37]. It indicates that managers with higher abilities are expected to develop effective plans and would like to take risks, helping to promote the entrepreneurial orientation and ensuring that research-oriented employees maximize their true potential to drive the implementation of digital transformation. Therefore, the following hypothesis is proposed:

**Hypothesis 1.** *Managerial ability is positively related to digital transformation*.

### 2.2 The moderating effect of historical aspiration shortfall

Performance shortfall is a noteworthy business stress within a firm, indicating a state of poor business conditions, decreasing performance, and loss of profits compared to the historical operating performance of the enterprise. The behavioral theory of the firm accentuates the processes of performance evaluation, search, and decision-making within organizations, with a specific emphasis on their impact on strategic decision-making [38]. At the core of this theory lies the notion that organizations shape their subsequent behavioral choices by evaluating the disparity between their present actual performance and their expected performance [38]. According to the behavioral theory of the firm, managers with different abilities will respond differently to the same business state. For example, when firms’ performances are lower than the aspiration level, competent managers are more motivated to accelerate digital transformation and positively construct and adjust their organizational resources, showing a more pronounced impact on digital transformation [39]. Hence, when firms encounter performance shortfalls, managers are compelled to pursue exploratory actions in order to restore performance to the desired aspiration level. Consequently, this prompts managers with greater capabilities to opt for more daring strategies of organizational change, thereby shedding light on the strategic impetus behind digital transformation.

The historical aspiration shortfall chooses a reference point based on the historical comparison, reflecting the tendency that decision-makers in management would compare current performances with their past performances [40]. When firms are experiencing a colossal performance aspiration shortfall, managerial abilities can enhance digital transformation. First, the historical aspiration shortfall refers to the problem of resource allocation or marketing strategy of the enterprise, which cannot meet the growing needs of the enterprise. Therefore, the downbeat performance will boost firms’ willingness to take risks, thus changing the current status [41]. Managers with more robust capabilities have a higher sense of risk-taking and are more motivated to implement digital transformation. In other words, outstanding managers prefer riskier transformation strategies to achieve extraordinary achievements and recognition in transforming decisions. Second, when the actual performance is lower than the historical aspiration level, the firm is in a slow growth stage, the internal resource allocation is insufficient, and it is difficult for the enterprise to obtain resources from external sources [42]. The firm will face a more uncertain environment, which increases the difficulty of the firm’s digital transformation.

Competent managers are usually biased in resources and risk perception, and they will show strong problem-search motivation to solve the current problem of performance shortfalls, deciding to take more risks and enhance their long-term control over digital transformation to facilitate the firm’s transformation strategy [43, 44]. The prospect of addressing historical aspiration shortfalls resonates with their achievement motivation, as goal aspirations serve as achievement targets that highly capable managers endeavor to attain [45]. Thus, underperformance will augment the risk preferences of managers with superior abilities. Specifically, managers possessing stronger capabilities will be motivated to undertake more substantial strategic risks (e.g., digital transformation), as historical aspiration shortfalls amplify the prominence of potential gains. Hence, we formulate the following hypothesis:

**Hypothesis 2.** *The historical aspiration shortfall moderates the relationship between managerial ability and digital transformation*, *such that the relationship will be stronger at a higher level of historical aspiration shortfall*.

### 2.3 The moderating effect of industrial competitiveness

The progression of enterprises is inextricably linked to the competitive milieu prevailing in their respective industries. This evolution is intrinsically intertwined with the collaborative and competitive dynamics that transpire among enterprise competitors within the market. The competitive backdrop of the industry, thus, assumes a pivotal role as an external determinant significantly impacting the decision-making processes related to innovation within enterprises. Drawing upon the behavioral theory of the firm, the external environment encompassing an enterprise exerts a discernible influence on the behavioral decision-making of its managerial cadre [46]. This perspective underscores that the exercise of managerial ability varies in accordance with the prevailing competitive landscape within the industry in which the enterprise operates. Thus, industrial competitiveness, denoting the level of competition among firms within the sector, stands as a pivotal metric regulating the contours of corporate strategic development.

As more enterprises opt for innovation and transformative initiatives to bolster their growth prospects, the competitive dynamics within the industry invariably intensify. For instance, in an industry characterized by pronounced monopolization and a dearth of competitors, managers enjoy the latitude to dedicate a substantial portion of their time and resources to Research and Development (R&D) and innovation, without being unduly concerned about potential market entrants. Consequently, this environment enables enterprises to formulate enduring strategic objectives for long-term development. Conversely, in sectors characterized by heightened competition, wherein diverse enterprises vie for innovative breakthroughs, managers confront amplified pressures.

This heightened competition translates into a formidable challenge for managers, as they strive to sustain corporate profitability and stabilize the enterprise’s entrenched position within the industry. The mounting pressures impede their capacity to harness innovative capabilities, thereby diminishing the enterprise’s level of digital transformation [47]. This heightened competition translates into a formidable challenge for managers, as they strive to sustain corporate profitability and stabilize the enterprise’s entrenched position within the industry. The mounting pressures impede their capacity to harness innovative capabilities, thereby diminishing the enterprise’s level of digital transformation.

Digital transformation within an organization is inherently an innovative undertaking, necessitating judicious managerial ability. When the external industrial competitive landscape is relatively benign, proficient managers, buoyed by their farsighted strategic vision and an acute sense of contemporary innovation, tend to exhibit a proclivity for risk in their strategic choices, all in pursuit of the goal of digital transformation [48]. Conversely, in industries grappling with intense competition, the external industrial milieu exerts greater pressure on managers, compelling them to concentrate on sustaining the organization’s extant growth trajectory. The relentless competition within such industries undermines the managerial capacity to cultivate an efficient production milieu, thereby jeopardizing the organization’s ability to retain its existing market share [49]. Consequently, in this milieu of heightened industry competition, enterprises are compelled to engage in fierce rivalry for pivotal resources and market shares, which in turn reduces their investment to implement digital transformation strategies.

Thus, elevated industrial competitiveness may adversely impact enterprises, leading to a concomitant reduction in their ability to execute digital transformation strategies. Within such a scenario, the external pressures exerted by industrial competitiveness tend to induce managers toward more conservative decision-making, thereby diminishing their inclination toward digital transformation initiatives. Hence, we formulate the following hypothesis:

**Hypothesis 3.** *The industrial competitiveness moderates the relationship between managerial ability and digital transformation*, *such that the relationship will be weakened at a higher level of industrial competitiveness*.

**Fig 1:**
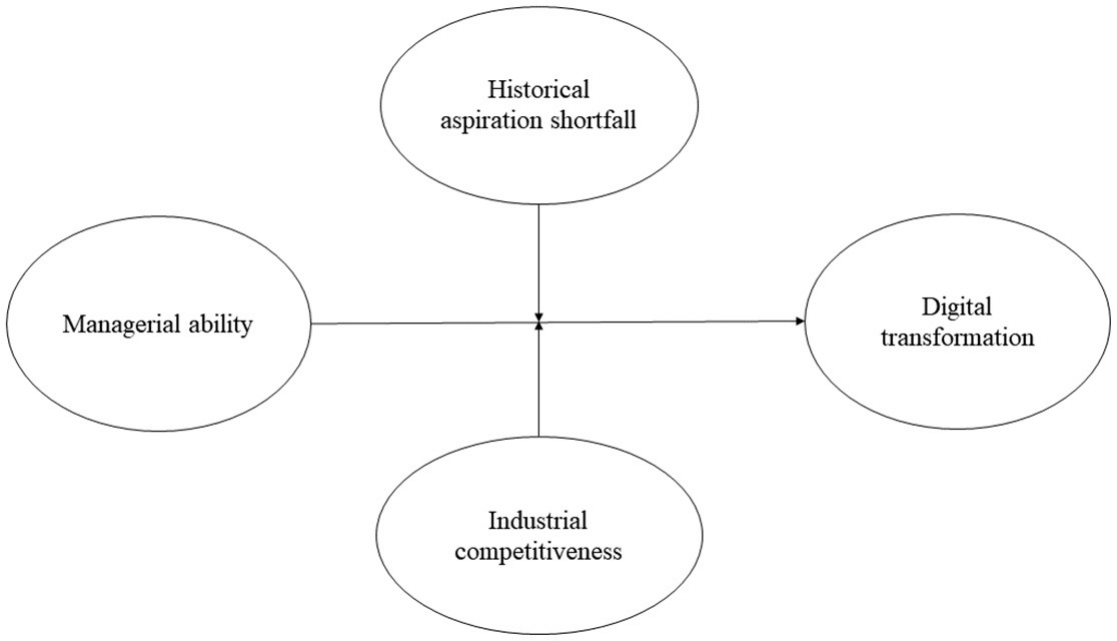


In order to investigate the hypotheses outlined above, Fig 1 presents the research model based on the theoretical analysis.

## 3. Data and methodology

### 3.1 Data and samples

This study chose Chinese A-share listed enterprises between 2009 and 2019 as samples from the China Stock Market Accounting Research (CSMAR) database and searched related digital transformation information from prospectuses, annual reports, announcements, and Juchao information website (www.cninfo.com.cn/new/index). The CSMAR database is a professional data set widely used in studies on corporate governance, including governance information (e.g., executive changes, board committee information, and executive demographic information) from all the listed enterprises in China [50, 51].

This study excluded data pertaining to underperforming entities, as well as stocks subject to special treatment. Additionally, data from the financial and real estate sectors were omitted, along with firms possessing missing data on specific variables, those with a leverage ratio exceeding 1, and those that issued B-share stocks to foreign investors. Moreover, to mitigate the potential impact of outliers, continuous variables were subjected to winsorization at the top and bottom 1% [50, 51]. The resulting sample encompassed 14,873 observations.

### 3.2 Dependent variable

Prior studies have suggested various measures on the variable of digital transformation (DT), defined as the degree of a firm’s digital transformation. Thus, this study constructed the DT index of listed firms via machine learning and text-mining technology. First, digital transformation involves the application of digital technology, which means firms use digital technology to improve the existing technical system and promote the digitization of the production system. These technology systems, such as artificial intelligence, blockchain, cloud computing, and big data, constitute the core underlying technology architecture for digital transformation [1]. Second, combined with previous quantitative analysis, this study condensed the role of digital transformation as a strategic behavior [52, 53]. Hence, we crawled and matched the data related to digital transformation from listed firms’ annual reports. The vocabulary in annual reports can display firms’ strategic characteristics and future tendencies, which is the possible development path guided by their preferred operational philosophies. Most firms disclose their implementation status of digital transformation in annual reports. Therefore, it is relatively feasible and scientific to portray the degree of digital transformation by considering the words related to digital transformation in firms’ annual reports as a proxy indicator of digital transformation.

Regarding the technical implementation of variable design, this study followed prior research and collected the annual reports from Chinese A-shared listed firms in the Shanghai and Shenzhen exchanges [54]. We collated and extracted all textual content through a Python program, establishing a data pool for subsequent feature word screening. According to the information from the academic and individual fields, we confirmed keywords related to digital transformation. First, based on the influential literature about digital transformation, we summarized these keywords related to digital transformation. We created a critical dictionary for text retrieval, including digital transformation, big data, cloud computing, blockchain, and artificial intelligence [1, 2, 8]. Second, the text of listed firms’ annual reports was extracted via Python to search, match, and count word frequencies based on these tools, such as Jieba word division and deactivation word list. Finally, we categorized and aggregated the frequencies of keywords and formed the final summed word frequencies. Because of the typical right bias owned by the data, we executed a logarithmic processing in which we exploited In (DT keyword disclosure + 1) to measure digital transformation, obtaining the overall indicators of digital transformation.

### 3.3 Independent variable

Managerial ability (MA) was measured by the relative level of firms’ output efficiency in the industry through the data envelopment analysis [14]. We adopted the Tobit model to control annual and industry dummy variables to measure management capability.

First, we used operating revenue (Sales) as the consequence variable and used the R&D investment (R&D), Goodwill, net operating lease expense (Netol), cost of doing business (CoGS), net fixed assets (PPE), intangible assets (Intangible), and selling and administrative expenses (SG&A) as input variables.

Specifically, firm efficiency is based on Data Envelopment Analysis (DEA) using seven inputs and one output: Inputs: net fixed assets (PPE) at the beginning of the fiscal year. An alternative way to access similar fixed assets is through operating leases across many sectors (airlines, retailers, hotels). The structure of operating leases allows firms to exclude the asset (and related debt) from their balance sheet, although these assets will generate revenues; thus, we estimate their capitalized value. CoGS is calculated through the cost of goods sold. SG&A is calculated through selling, general, and administrative expenses. Net Operating Leases (Netol) is the discounted present value of the next five years required operating lease payments, available in the firm’s footnotes to the financial statements. Net research and development (R&D) investment (RD), which is not reported as an asset on the balance sheet. We use a five-year capitalization period of research and development expense (XRD) [55], where the net value (net of amortization) is ${RD}_{cap}=\sum_{t=-4}^{0}(1+0.2t)\times {RD}_{exp}$. Thus, research and development expenditures from five years back receive a weight of 0.2 (they were already amortized 80%), those from four years back receive a weight of 0.4 (amortized 60%), etc., with the prior year’s research and development (t = -1) receiving full weight. Purchased goodwill (GoodWill) is the excess of the purchase price for a business acquisition over the amounts allocated to other separately identifiable assets and liabilities; and other acquired and capitalized intangibles (Intangible). Output is operating revenue (SALE).

Then, the DEA method was exploited to measure the optimal input-output efficiency of the sub-industries, yielding a firm-year ratio (θ) relative to the industry’s optimal level.


$$Max_{v}\theta =Sales/(\nu_{1}PPE+\nu_{2}Intangible+\nu_{3}GoodWill+\nu_{4}R\&D+\nu_{5}CoGS+\nu_{6}SG\&A+\nu_{7}Netol)$$ (1)

Second, a Tobit model was developed, including free cash flow (FCF), firm size (Size), corporate listing date (Age), market share (Marketshare), the degree of diversification (HHI), and the presence of overseas subsidiaries (FC), to remove factors affecting the firm’s productivity and obtain residuals indicating the strength of managerial ability.


$$\theta =\alpha_{0}+\alpha_{1}Size+\alpha_{2}Markershare+\alpha_{3}FCF+\alpha_{4}Age+\alpha_{5}HHI+\alpha_{6}FC+Year+Ind+\varepsilon$$ (2)

The methodology above calculated the operational efficiency of listed companies through a large sample, considering inter-group correlations, and more accurately estimating management ability by clustering at the firm level to adjust for standard errors.

### 3.4 Moderating variables

The behavioral theory of the firm focuses on organizational performance evaluation, search, and decision-making processes, and how these processes affect strategic organizational decisions. The theory suggests that the organization determines strategic choices by evaluating the current and expected performances. When the current performance falls short of the expected performance, managers will deem the current performance, which is lower than the expected performance, as a loss status of the organization; and this loss status spurs managers to engage in exploratory behavior to restore organizational performance to the aspiration-level [56]. Moreover, extant literature concentrated on reference points for performance aspiration levels: historical aspirations, which compare performance with the firm’s record. Firms evaluate their current performance relative to these reference points, and utilize this data to modify strategic decisions. Therefore, supported by the behavioral theory of the firm, this study measures historical aspiration shortfall.

For firms’ performance aspirations, we followed previous studies to compare past performances owned by target firms (i.e., historical aspiration). To measure performance, we calculated a firm’s return on assets (ROA) as its net income divided by the average of its beginning and ending total assets in a specific fiscal year. A firm’s historical aspiration level was defined as the firm’s ROA in the previous year.

An enterprise’s historical aspiration refers to the difference between its actual performance in the current year and its historical aspiration level in the current year. For the measurement of historical aspiration level, the classical recursive measure was exploited [57]. Since this study relates to the moderating effect of the difference between actual and aspiration performances, the moderating variable was lagged by one period concerning the dependent variable. The formula is as follows:

$$A_{i,t}=\alpha_{1}P_{i,t-1}+\left(1-\alpha_{1}\right)A_{i,t-1}$$ (3)

*A*_*i*,*t*_ denotes the historical aspiration level of enterprise *i* in year t and *P*_*i*,*t*–1_ denotes the actual performance of enterprise *i* in year t-1. *α*_1_ denotes the weight between the actual performance in the previous period and the aspiration level in the previous period, and takes a value range of [0, 1]. This study chose *α*_1_ = 0.6 for the historical aspiration level measure [57]. It should be noted that *A*_*i*,0_ is the historical aspiration level of enterprise *i* in period 0, which is replaced by the actual performance in period 0. Furthermore, we measured the variable of performance historical aspiration shortfall (HAS) by taking the absolute value of the difference if the enterprise’s actual performance is lower than the historical aspiration level, otherwise taking 0 [58].

Industrial competitiveness (IC) in this study is measured using the Herfindahl-Hirschman Index (HHI) for each segmented industry in the current year. The methodology employed draws from measurement methods outlined by Haveman et al. and Jia et al. [59, 60]. First, the manufacturing industries are categorized based on the two-digit industry codes as per the China Securities Regulatory Commission (2012 version). Second, the market share of each enterprise is determined by assessing the revenue of all firms within each sub-sector. The final step involves calculating the square sum of the market share of all enterprises operating within the sub-sector for the given year. This computation yields the Herfindahl-Hirschman Index (HHI) for that sub-sector in that specific year. For the purpose of facilitating the interpretation of the subsequent empirical findings, the HHI index is subjected to negative processing (multiplying by -1). This processing results in the Industrial Competitiveness (IC) index that is utilized in the model testing. It is important to note that a larger IC index denotes a stronger level of industrial competitiveness. In summary, the IC index, derived from HHI calculations and subjected to negative processing, serves as a crucial metric in assessing the strength of industrial competitiveness within each segmented industry, thereby enabling its incorporation into the model for empirical analysis.

### 3.5 Control variables

We controlled for several other factors that might influence the digital transformation of enterprises. *Size*, which refers to firm size, is the natural logarithm of an enterprise’s total asset. *Age*, which refers to firm age, is measured from an enterprise’s founding date. *Board* is measured from the logarithm of the number of board members. *Inde* is calculated from the proportion of sole directors. *Dualiy* equals one if the CEO and board director are the same person and zero otherwise. *InsInv* is measured from the Shareholding of institutional investors. *ShBa* is estimated from the sum of the shareholdings of the top ten largest shareholders. Other financial indicators include *Growth* (growth rate of total assets), *Cash* (ratio of free cash flow to total assets). We also use the number of firm and year dummies to control for different enterprise innovations and the impact of year and industry on enterprise survival, respectively. The variables are defined as shown in Table 1.

**Table 1:** Definitions of key variables.

Variable type	Variable symbols	Variable name	Variable definitions
Dependent variable	*DT*	Digital transformation	The number of disclosures on the keyword "digital transformation" in the annual reports of listed companies is added by one and taken as the natural logarithm
Independent variable	*FEC*	Firm efficiency	Firm efficiency, based on Data Envelopment Analysis (DEA), includes seven inputs and one output: Inputs: net property, plant, and equipment (PPE); cost of goods sold (CoGS); selling, general, and administrative expenses (S&GA), capitalized operating leases calculated as the discount present value of the next five years of required operating lease payments using a discount rate of 10 percent; net research and development (R&D) costs; purchased goodwill (GoodWill); net operating lease expense (Netol), and other acquired and capitalized intangibles (Intangible); Output: revenues (Sales)
	*MA*	Managerial ability	The DEA and Tobit methods were used in two stages to calculate
Moderating variables	*HAS*	Historical aspirations shortfall	The absolute value of the difference is taken when the enterprise’s actual performance falls below the historical aspiration level; otherwise, it is set to zero
	*IC*	Industrial competitiveness	The Herfindahl-Hirschman Index (HHI) for each industry segment in the current year is utilized and is considered in its negative form
Control variables	*Size*	Business size	Total assets at the end of the period plus one, rounded to the nearest natural logarithm
	*Age*	Age of business	Number of years of incorporation plus one, rounded to the nearest natural logarithm
	*Board*	Board size	The logarithm of the number of board members
	*Inde*	Proportion of sole directors	Number of independent directors/total number of board members
	*Dualiy*	A CEO with both positions	1 when the CEO is also the Chairman, 0 otherwise
	*InsInv*	Institutional Investor Holdings	Shareholding of institutional investors
	*ShBa*	Concentration of shareholding	The sum of the shareholdings of the top ten largest shareholders of the Company
	*Growth*	Company Growth	The growth rate of total assets at the end of the year
	*Cash*	Free cash flow	Net cash flow from operating activities/total assets
	*Year*	Year fixed effects	Year dummy variables
	*Industry*	Industry fixed effects	Industry dummy variables

### 3.6 Model specification

To test the hypothesis 1, we proposed the regression model as follows:

$$DT_{i,t+1}=\alpha_{0}+\alpha_{1}\times MA_{i,t}+\alpha_{k}\sum Control_{i,t}+\varepsilon_{i,t}$$ (4)

Then, to test the hypothesis 2 and 3, we proposed the regression model to test the moderating effect.


$$DT_{i,t+1}=\beta_{0}+\beta_{1}MA_{i,t}+\beta_{2}\times HAG_{i,t}+\beta_{3}MA_{i,t}\times HAG_{i,t}+\beta_{4}\times IC_{i,t}+\beta_{5}MA_{i,t}\times IC_{i,t}+\beta_{k}\sum Control_{i,t}+\varepsilon_{i,t}$$ (5)

The subscript *i* represents the *i*th listed enterprise, and *t* represents every year. This study performed model estimation for fixed-effects regression, controlling for industry, and year-fixed effects, and denotes the summation of the model’s control variables, including the enterprise level and financial variables, and the random error term.

## 4. Empirical results

### 4.1 Descriptive statistics

Table 2 shows the results of descriptive statistics for each variable. Digital transformation has a mean value of 3.3500, a standard deviation of 1.3527, a minimum value of 0, and a maximum value of 7.5022, indicating a clear trend in the overall digital transformation of listed companies in China, but the digital transformation of enterprises varies significantly between different listed enterprises, reflecting differences in transformation capabilities. Managerial ability has a maximum value of 0.3903 and a minimum value of 0, with a mean value of -0.0057 and a standard deviation of 0.1513, indicating that managerial ability varies between enterprises. In terms of moderating variables, the mean value of historical aspirations shortfall was 0.0181 with a standard deviation of 0.0411, while the mean value of industrial competitiveness was -0.1148 with a standard deviation of 0.1241, indicating differences in historical aspiration shortfall and industrial competitiveness faced by different enterprises. In addition, the mean value of the firm efficiency indicator in the first stage of the DEA model is 0.6304 with a standard deviation of 0.1677, which is similar to existing research papers [13].

**Table 2:** Descriptive summary.

Variables	Obs	Mean	SD	Min	Med	Max
*DT*	14873	3.3500	1.3527	0.0000	3.2581	7.5022
*FEC*	14873	0.6304	0.1677	0.3110	0.5971	1.0000
*MA*	14873	-0.0057	0.1512	-0.2844	-0.0330	0.3903
*HAS*	14873	0.0181	0.0411	0.0000	0.0003	0.2790
*IC*	14873	-0.1148	0.1241	-0.7652	-0.0751	-0.0159
*Size*	14873	22.0660	1.2345	19.9094	21.8791	26.0627
*Age*	14873	2.7930	0.3612	1.6094	2.8332	3.4657
*Board*	14873	2.2406	0.1742	1.7918	2.3026	2.7726
*Inde*	14873	0.3757	0.0538	0.3333	0.3529	0.5714
*InsInv*	14873	0.4179	0.2499	0.0019	0.4350	0.9033
*ShBa*	14873	0.7456	0.6139	0.0293	0.5813	2.8486
*Dualiy*	14873	0.2906	0.4541	0.0000	0.0000	1.0000
*Growth*	14873	0.1678	0.3038	-0.2988	0.0949	1.8739
*Cash*	14873	0.0462	0.0661	-0.1457	0.0445	0.2339

### 4.2 Regression results

Table 3 represents the results from the fixed effects regression. Model 1 provides a base model without control variables. Model 2 includes the main effect of managerial ability and the digital transformation of enterprises. The results of Model 1 and 2 show that the estimated coefficients of managerial ability are significantly positive (coef = 0.1587, p<0.01, coef = 0.1273, p<0.05), which suggest that when the managerial ability is higher, it promotes a higher motivation to implement digital transformation and achieve long-term stable enterprise growth. The results above support managerial ability’s positive contribution to digital transformation, which supports Hypothesis 1.

**Table 3:** Managerial ability and digital transformation.

	Model 1	Model 2
	DT	DT
*MA*	0.1587***	0.1273**
	(3.6600)	(2.9999)
*Size*		0.1748***
		(5.2785)
*Age*		-0.2387***
		(-4.1089)
*Board*		0.2079***
		(5.2047)
*Inde*		-0.1445**
		(-2.3698)
*InsInv*		0.0634
		(1.1004)
*ShBa*		0.0245**
		(2.6092)
*Dualiy*		0.0266**
		(2.2653)
*Growth*		0.0649***
		(3.8449)
*Cash*		0.1930
		(1.7686)
*Constant*	2.1482***	-1.5399**
	(11.4561)	(-2.4500)
*Industry FE*	Yes	Yes
*Year FE*	Yes	Yes
*N*	14873	14873
*Within-R* ^*2*^	0.3974	0.4169

Notes:

***Significant at p<0.01,

**Significant at p<0.05,

*Significant at p<0.1.

This table reports the results of regression coefficient estimates of the relation between managerial ability and digital transformation. The sample consists of CSMAR enterprises for which financial and corporate governance data are available in 2009–2019. All the variables are defined in Table 1. The results are estimated by the fixed-effects model considering different control variables [14]. Year dummy and Industry dummy variables are included in both regressions. The t-statistics, reported in parentheses, are obtained after considering standard errors.

### 4.3 Moderating effects

Table 4 reports the results of estimating the moderation effects of historical aspiration shortfalls and industrial competitiveness on the relationship between managerial ability and enterprise digital transformation. According to Driscoll and Kraay [61], the results were estimated by the fixed-effects model by considering different control variables and standard errors. The results of Model 1 and Model 2 in Table 4 show that the estimated coefficients of MA×HAS are significantly positive (coef. = 2.6516, p<0.05; coef. = 2.3806, p<0.05), indicating that historical aspiration shortfall has a moderating effect on the relationship between managerial ability and the digital transformation. When historical aspiration shortfall increases, it enhances the effect of managerial ability on digital transformation. This result indicates that managers make strategic transformation decisions based on historical business dilemmas, which leads to more digital transformation behaviour and supports Hypothesis 2. Also, the results of Model 3 and Model 4 show that the estimated coefficients of MA×IC are significantly negative (coef. = -0.4903, p<0.1; coef. = -0.8077, p<0.05), indicating that industrial competitiveness has a moderating effect on the relationship between managerial ability and digital transformation, thus supporting Hypothesis 3. In conclusion, from the perspective of a firm, it becomes evident that managerial ability serves as a critical foundation for a company’s digital transformation, particularly when confronted with internal performance challenges. Finally, we conducted an examination of the integrated interaction effects in Model 5. The hierarchical models enable the discernment of the distinct contributions of MA and its associated interaction terms.

**Table 4:** Testing moderating effects of historical aspiration shortfall and industrial competitiveness.

	Model 1	Model 2	Model 3	Model 4	Model 5
	DT	DT	DT	DT	DT
*MA*	0.1108***	0.1016**	0.1534***	0.1164**	0.1002**
	(3.3082)	(2.6534)	(3.5213)	(2.5101)	(2.6523)
*HAS*	-0.5312***	-0.3590***			-0.3592***
	(-3.4004)	(-3.3501)			(-3.3162)
*MA x HAS*	2.6516**	2.3806**			2.3868**
	(2.4615)	(2.3596)			(2.3679)
*IC*			0.0675	0.0742	0.0538
			(0.8111)	(0.7990)	(0.4438)
*MA x IC*			-0.4903*	-0.8077**	-0.3509
			(-1.9384)	(-2.9225)	(-0.8380)
*Size*		0.1435***		0.1767***	0.1440***
		(3.9250)		(5.2013)	(3.9019)
*Age*		-0.2224***		-0.2418***	-0.2225***
		(-5.7812)		(-4.2404)	(-5.6962)
*Board*		0.2038***		0.2083***	0.2041***
		(4.0872)		(5.1330)	(4.1630)
*Inde*		-0.2117		-0.1504**	-0.2135
		(-1.5766)		(-2.4979)	(-1.6096)
*InsInv*		0.1522**		0.0661	0.1537**
		(2.5943)		(1.1662)	(2.7132)
*ShBa*		0.0214**		0.0245**	0.0213**
		(2.3928)		(2.6513)	(2.3695)
*Dualiy*		0.0265*		0.0271**	0.0266*
		(1.9971)		(2.3046)	(2.0062)
*Growth*		0.0654***		0.0642***	0.0652***
		(6.2793)		(3.7257)	(6.2081)
*Cash*		0.0817		0.1964	0.0824
		(0.7895)		(1.7703)	(0.7830)
*Constant*	1.7553***	-1.3185	2.1686***	-1.5504**	-1.3121
	(12.1072)	(-1.5118)	(10.5698)	(-2.4883)	(-1.4946)
*Industry FE/Year FE*	Yes	Yes	Yes	Yes	Yes
N	14873	14873	14873	14873	14873
Within R^2^	0.3891	0.4002	0.4039	0.4172	0.4003

Notes:

***Significant at p<0.01,

**Significant at p<0.05,

*Significant at p<0.1.

### 4.4 Robustness tests

In our efforts to enhance the robustness of our findings, we initially replace the measurements of managerial ability and digital transformation (see Table 5). Specifically, we have substituted the proxy for managerial ability with managerial ability rank (MA Rank), which is a ranking of managerial ability provided by Demerjian [14]. This change aims to mitigate potential measurement errors in the assessment of managerial ability scores. The outcomes of Model 1 and Model 2, as presented in Table 5, reveal a significant and positive association between managerial ability rank and digital transformation after accounting for firm-fixed effects and year-fixed effects. Furthermore, Model 3 and Model 4, which utilize alternative measures of digital transformation, as indicated in Table 5, consistently demonstrate a positive impact of managerial ability on the new digital transformation indicator.

**Table 5:** Replace the measurements of managerial ability and digital transformation.

	Model 1	Model 2	Model 3	Model 4
	DT	DT	DT_New	DT_New
*MA Rank*	0.0094**	0.0057*		
	(2.6951)	(2.2514)		
*MA*			0.1414***	0.1445***
			(6.4891)	(6.6552)
*Size*		0.1827***		-0.0274***
		(5.3862)		(-7.4523)
*Age*		-0.2774***		-0.1096***
		(-4.9252)		(-9.6179)
*Board*		0.2110***		0.0328***
		(4.8692)		(4.4145)
*Inde*		-0.1700**		0.0501
		(-2.7258)		(1.6912)
*InsInv*		0.0551		0.0422**
		(0.9046)		(2.3876)
*ShBa*		0.0174		0.0006
		(1.7842)		(0.2028)
*Dualiy*		0.0232		0.0022
		(1.8164)		(0.6329)
*Growth*		0.0647***		-0.0056
		(4.2697)		(-1.1368)
*Cash*		0.2096*		-0.0444**
		(1.9970)		(-2.8134)
*Constant*	2.1332***	-1.6154**	0.5887***	1.3210***
	(11.4662)	(-2.4895)	(12.2405)	(12.8594)
*Industry FE/Year FE*	Yes	Yes	Yes	Yes
N	14873	14873	14873	14873
Within R^2^	0.3972	0.4105	0.0684	0.0900

Notes:

***Significant at p<0.01,

**Significant at p<0.05,

*Significant at p<0.1.

Secondly, we replace the estimation method and standard errors. In the robustness test, the regression analysis is carried out utilizing a high-dimensional fixed effects model, drawing from the research conducted by Gormley and Matsa et al. [62]. Additionally, in accordance with Imbens and Kolesár [63], the analysis employs clustered robust standard error calculations. The empirical results presented in Table 6 affirm that the re-examination corroborates our conclusions.

**Table 6:** Replace the estimation method and standard errors.

	Model 1	Model 2	Model 3	Model 4
	DT	DT	DT	DT
*MA*	0.1587**	0.1301*	0.1600**	0.1273*
	(1.9998)	(1.6551)	(2.0608)	(1.6533)
*Size*		0.1837***		0.1748***
		(6.8818)		(6.6805)
*Age*		-0.2815*		-0.2387
		(-1.8467)		(-1.5945)
*Board*		0.2111**		0.2079**
		(2.2754)		(2.2565)
*Inde*		-0.1697		-0.1445
		(-0.6853)		(-0.5912)
*InsInv*		0.0497		0.0634
		(0.4942)		(0.6422)
*ShBa*		0.0172		0.0245
		(0.5473)		(0.7993)
*Dualiy*		0.0231		0.0266
		(0.9482)		(1.1123)
*Growth*		0.0621***		0.0649***
		(3.3975)		(3.6004)
*Cash*		0.1891*		0.1930*
		(1.7940)		(1.8847)
*Constant*	3.4276***	-0.3161	2.1476***	-1.5399**
	(4992.6803)	(-0.4246)	(6.1004)	(-1.9812)
*Industry FE/Year FE*	Yes	Yes	Yes	Yes
N	14421	14421	14873	14873
Adj.R^2^ /Within.R^2^	0.8520	0.8551	0.4038	0.4169

Notes:

***Significant at p<0.01,

**Significant at p<0.05,

*Significant at p<0.1.

In the third robustness test, as presented in Table 7, we adopt the higher-order joint fixed effect models proposed by Moser and Voena, which effectively controls for the indicator of Industry FE×Year FE [64]. Because the utilization of two-way fixed effect models, encompassing both time and industry dimensions, in regression analysis has been a conventional practice. However, it may be argued that this approach, while flexible, might not provide a sufficiently rigorous control for endogeneity concerns. Furthermore, it is important to acknowledge that firms’ digital transformation behavior is intricately linked with broader financial shocks [65]. Within the time series examined in this study, a salient financial event was the 2015 stock market crash in China. Nonetheless, quantifying the impact of such events using specific variables can be challenging. To address this issue, we have taken measures to remove the influence of the Chinese stock market crash crisis from our analysis [65]. Subsequently, we have excluded the samples from the years immediately following the crisis due to the profound and lasting effects it had on the financial landscape. The results presented in Table 7 demonstrate that, after controlling for joint higher-order fixed effects and eliminating the impact of the Chinese stock market crash, there exists a statistically significant and positive association between managerial ability and digital transformation.

**Table 7:** Replace the fixed effect models and data.

	Model 1	Model 2	Model 3	Model 4
	DT	DT	DT	DT
*MA*	0.1510**	0.1293**	0.1233***	0.1234**
	(2.9055)	(2.6328)	(4.1388)	(3.0202)
*Size*		0.1995***		0.1951***
		(6.1425)		(6.5982)
*Age*		-0.3203***		-0.2743***
		(-5.3459)		(-4.8829)
*Board*		0.2087***		0.2451***
		(3.9804)		(5.3010)
*Inde*		-0.1892**		-0.1057
		(-2.2874)		(-0.9049)
*InsInv*		0.0361		0.0282
		(0.6154)		(0.4472)
*ShBa*		0.0145		0.0237
		(1.5093)		(1.3203)
*Dualiy*		0.0206		0.0161
		(1.6158)		(1.5494)
*Growth*		0.0525***		0.0438
		(3.5918)		(1.4280)
*Cash*		0.1886		0.1052
		(1.6608)		(0.8148)
*Constant*	3.0302***	-0.9374	2.2385***	-1.8510***
	(13.1756)	(-1.2927)	(10.5364)	(-3.5526)
*Industry FE/Year FE*	No	No	Yes	Yes
*Industry FE×Year FE*	Yes	Yes	No	No
N	14873	14873	11108	11108
Within R^2^	0.4083	0.4223	0.4250	0.4377

**Fig 2:**
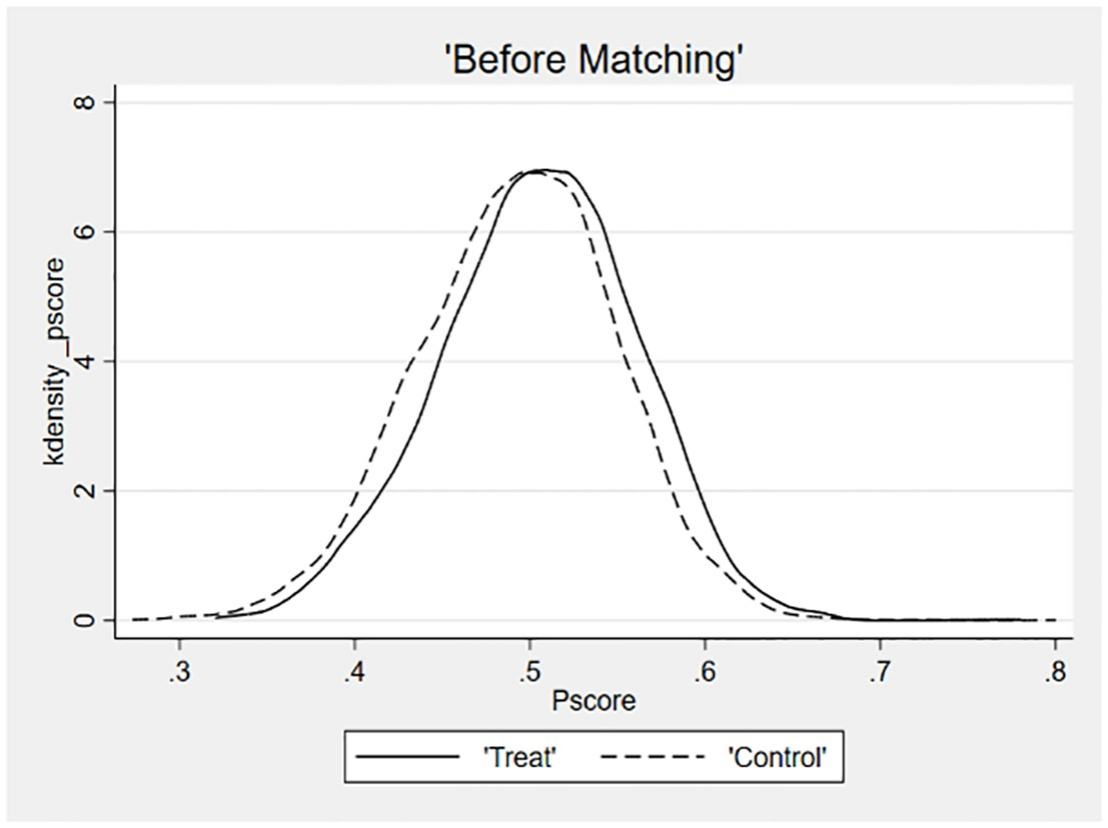


**Fig 3:**
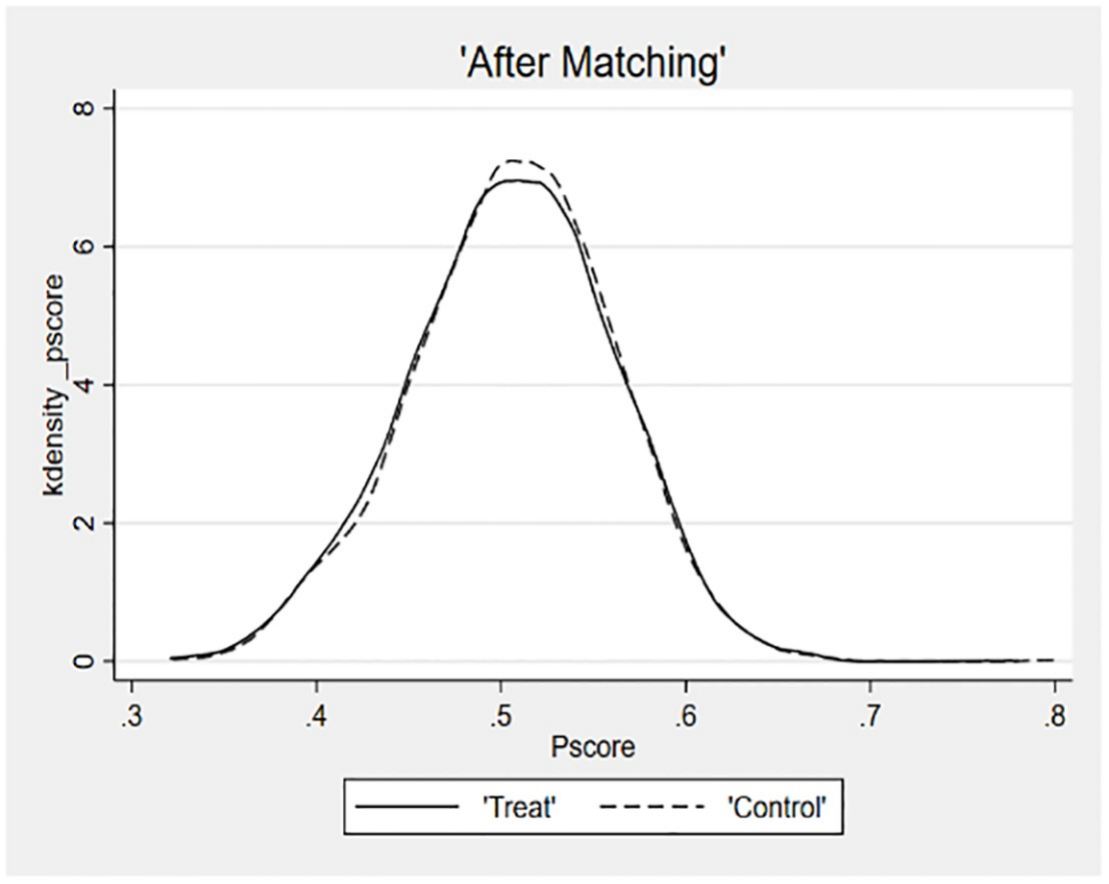


The fourth robustness test employs the Propensity Score Matching (PSM) method, as illustrated in Table 8. The kernel density plots are utilized to visually represent the effects of sample matching, thereby helping to mitigate potential measurement errors and standard errors [61]. Following the procedure outlined by Custódio et al., the specific steps of the PSM test were as follows [66]. The samples of higher managerial ability are divided into two groups according to their median levels, and 11, 533 samples are obtained by 1-to-1 nearest neighbour matching in the control group of samples of lower managerial ability. Figs 2 and 3 show that the difference between the two groups of samples after matching is reduced, indicating a good match. The matched sample is further tested, which continuous to support the research hypotheses.

**Table 8:** The propensity score matching method (PSM) test result.

	Model 1	Model 2
	DT	DT
*MA*	0.1547**	0.1237*
	(2.7809)	(1.9773)
*Size*		0.1717***
		(5.7854)
*Age*		-0.3221***
		(-12.3441)
*Board*		0.2383***
		(4.4125)
*Inde*		-0.1986**
		(-2.5682)
*InsInv*		0.0222
		(0.3526)
*ShBa*		0.0525**
		(2.4084)
*Dualiy*		0.0363***
		(3.3284)
*Growth*		0.0678**
		(3.1491)
*Cash*		0.2087
		(1.4559)
*Constant*	2.1786***	-1.3096*
	(10.4923)	(-2.1383)
*Industry FE/Year FE*	Yes	Yes
N	11533	11533
Within R^2^	0.3959	0.4104

Notes:

***Significant at p<0.01,

**Significant at p<0.05,

*Significant at p<0.1.

The fifth robustness test employs the Heckman two-stage regression test. The results show a strong and consistent association between managerial ability and digital transformation. However, our findings might be vulnerable due to endogeneity concerns. For example, enterprises with high digital transformation can hire more able managers, indicating a reverse causality problem. Digital transformation and managerial ability also may be determined simultaneously, by unobserved risk factors. In the analysis before, we calculated enterprise and time-fixed effects in the regressions to control for the time-invariant and time-varying factors that may affect both the digital transformation and managerial ability. However, it is different to eliminate the endogeneity biases in the study. Therefore, to mitigate the self-selection effects between managerial ability and the transformation of enterprises, a two-stage regression is conducted using the managerial ability, which is common to take the industry mean in the previous year as an instrumental variable in the existing literature [67]. It is because the managerial capability of an individual enterprise is usually correlated with the previous year’s industry average managerial capability profile for the industry in which it operates (a collection of enterprises engaged in similar production activities), but changes in the degree of enterprise’s digital transformation are unlikely to affect the historical average managerial capability profile in the industry. Table 9 shows that the Inverse Mills Ratio (IMR) is insignificant, indicating the endogeneity problem is not a problem in this study, and our findings are robust.

**Table 9:** Heckman two-stage test.

	Model 1	Model 2
	MA_X	DT
*IV*	6.0680***	
	(6.6575)	
*MA*		0.1278***
		(3.8165)
*IMR*		-0.0598
		(-0.6900)
*Size*	-0.0951***	0.1440***
	(-9.6076)	(3.3876)
*Age*	0.2528***	-0.2438***
	(8.0851)	(-6.3124)
*Board*	-0.1129	0.2221***
	(-1.5788)	(3.5230)
*Inde*	-1.0892***	-0.1539
	(-4.9461)	(-0.8621)
*InsInv*	0.2321***	0.1714**
	(5.1493)	(2.9035)
*ShBa*	-0.0746***	0.0231**
	(-4.4604)	(2.2802)
*Dualiy*	-0.0242	0.0233
	(-1.0905)	(1.4914)
*Growth*	0.3564***	0.0632**
	(10.3490)	(2.7657)
*Cash*	2.0612***	0.0403
	(13.4345)	(0.2246)
*Constant*	1.9380***	-1.7333
	(6.7678)	(-1.8044)
*Industry FE*	Yes	Yes
*Year FE*	Yes	Yes
*N*	14873	11706
*Pseudo -R* ^*2*^ */Within-R* ^*2*^	0.0818	0.4023

Notes:

***Significant at p<0.01,

**Significant at p<0.05,

*Significant at p<0.1.

In the sixth robustness test, as shown in Table 10, this study employs a two-stage least squares method to investigate the potential endogenous issue related to reverse causality between managerial ability and digital transformation. Leveraging insights from existing literature, we utilize lagged managerial ability (IV) as an instrumental variable [67]. The estimated coefficient for managerial ability (MA) remains highly significant at the 1% level across regression models that include all control variables or exclude variables prone to multicollinearity issues. These robust results lend support to our initial findings, underscoring the pivotal role of exceptional managerial ability in facilitating the effective implementation of digital transformation.

**Table 10:** Two-stage least squares test.

	Model 1	Model 2
	First-Stage	Second-Stage
	MA	DT
*IV*	2.3152***	
	(8.6226)	
*MA*		4.4136***
		(4.2502)
*Size*	-0.0154***	-0.0267
	(-14.0844)	(-1.3636)
*Age*	0.0270***	-0.1666***
	(8.1654)	(-4.0160)
*Board*	-0.0173**	-0.2472***
	(-2.2012)	(-3.4304)
*Inde*	-0.1308***	0.2912
	(-5.4354)	(1.1374)
*InsInv*	0.0321***	0.0026
	(6.4494)	(0.0471)
*ShBa*	-0.0078***	0.0676***
	(-4.2678)	(3.6999)
*Dualiy*	-0.0047*	0.1481***
	(-1.9237)	(6.3731)
*Growth*	0.0472***	0.5686***
	(13.2858)	(9.2346)
*Cash*	0.2533***	0.3942
	(15.0684)	(1.2635)
*Constant*	0.4025***	3.3175***
	(12.8436)	(6.8834)
*Industry FE*	Yes	Yes
*Year FE*	Yes	Yes
*Kleibergen-Paap RK LM statistic*		64.789***
*Cragg-Donald Wald F statistic*		74.321
		(16.38)
*Kleibergen-Paap RK Wald F statistic*		66.102
*N*	11780	11780
*Adj-R* ^*2*^	0.2259	0.2067

Notes:

***Significant at p<0.010,

**Significant at p<0.05,

*Significant at p<0.1.

## 5. Further research

### 5.1 Group testing

The above relationships may vary across different natures of property rights and levels of corporate governance. Therefore, the samples were grouped into state-owned enterprises (SOEs) and non-SOEs for group testing according to the nature of ownership. Also, the samples were grouped for further testing according to the median levels of corporate governance. The results are displayed in Table 11.

**Table 11:** Group tests based on differences in the nature of property rights and levels of governance.

	Model 1	Model 2	Model 3	Model 4
	State-owned enterprises	Non-state enterprises	High level of governance	Low level of governance
	DT	DT	DT	DT
*MA*	0.2217***	0.0934*	0.1186**	0.1180
	(3.6222)	(2.1459)	(3.3358)	(1.7734)
*Size*	0.0393	0.2022***	0.1502***	0.1748***
	(1.5458)	(6.4313)	(6.9264)	(5.1111)
*Age*	-0.3351**	-0.4457***	-0.4089***	-0.4299***
	(-2.7517)	(-5.5091)	(-6.5565)	(-3.4690)
*Board*	0.1816**	0.2453***	0.0623	0.2901***
	(3.0373)	(4.4901)	(1.2915)	(3.8327)
*Inde*	-0.4110**	-0.0974	-0.5220***	0.2368
	(-2.9014)	(-0.9133)	(-6.8666)	(1.7168)
*InsInv*	0.3470***	0.0652	0.2314***	0.1341*
	(4.0315)	(0.9623)	(4.1051)	(1.8624)
*ShBa*	0.0043	0.0306*	0.0619*	-0.0325*
	(0.1411)	(2.0142)	(2.0831)	(-1.8554)
*Dualiy*	0.0030	0.0246	-0.0257*	0.0184
	(0.1159)	(1.2325)	(-2.2808)	(1.5359)
*Growth*	0.1057***	0.0400**	0.0560***	0.0512
	(3.8334)	(2.4786)	(5.1137)	(1.6756)
*Cash*	0.0867	0.1491	0.0626	0.2577
	(0.5576)	(1.6227)	(0.3299)	(1.2301)
*Constant*	1.8734***	-2.4040***	0.6966	-2.2808**
	(3.3369)	(-3.4619)	(1.0532)	(-3.0262)
*Industry FE*	Yes	Yes	Yes	Yes
*Year FE*	Yes	Yes	Yes	Yes
*N*	4807	10066	7487	7386
*Within-R* ^*2*^	0.3604	0.4349	0.3626	0.3832

Notes:

***Significant at p<0.01,

**Significant at p<0.05,

*Significant at p<0.1.

The results in Table 11 show that the regression coefficients for managerial ability are all significantly positive in the SOE and higher governance level samples, while they are less significant or no longer significant in the non-SOE and lower governance level samples. The findings suggest that the effect of managerial ability on digital transformation is more robust in the SOE and higher governance samples compared to the non-SOE and lower governance samples. The reason may be that state-owned enterprises with higher levels of governance pay more attention to managerial efficiency, which means they own more flexible and standardized governance systems as a guarantee for managers to exercise their decisions. In addition, state-owned enterprises have more advantages in accessing national policies and related resources, so managerial ability plays a more critical role in the strategic choices of digital transformation.

### 5.2 Mechanism test

The regression results above demonstrate that the stronger the managerial ability, the greater the degree of digital transformation. The following question is how managerial ability promotes digital transformation. We suggest that there may be a managerial incentive mechanism and an entrepreneurial orientation mechanism, which needs to be further analysed. To test whether managerial ability influences the level of digital transformation through the two mechanisms mentioned above, the managerial equity incentive (MI) mechanism is measured using the level of managerial shareholding and taking the natural logarithm. Managerial shareholding reflects the willingness to make digital transformation decisions, which measures the extent to which the enterprise supports the manager’s future decisions. Therefore, it needs to test whether managerial ability contributes to the enterprise’s digital transformation through the equity incentive mechanism. Then, the following methodology will be used to test entrepreneurial orientation (EO) mechanisms, using annual R&D expenditure as a percentage of sales revenue and annual net cash flow from investing activities as a percentage of sales revenue to measure managerial entrepreneurial orientation (EO) [68].

Referring to Rodriguez & Nieto [69], we construct the following model [70]. *Y* means the level of digital transformation of the enterprise, *M* represents the mediating variable, *X den*otes managerial ability, and *Control* denotes the control variable. Similar to column 1, *C*_*1*_ *~ C*_*3*_ are constant terms, the *α*, *δ* and *γ* are regression coefficients, and *ε*_*it*_ are the random error term.


$$Y=C_{1}+\alpha_{1}X+\alpha \times \sum Control_{i,t}+\sum Year+\sum Industry+\varepsilon_{it}$$ (6)

$$M=C_{2}+\delta_{1}X+\delta \times \sum Control_{i,t}+\sum Year+\sum Industry+\varepsilon_{it}$$ (7)

$$Y=C_{3}+\gamma_{1}X+\gamma_{2}M+\gamma \times \sum Control_{i,t}+\sum Year+\sum Industry+\varepsilon_{it}$$ (8)

Firstly, we analyzed equity incentives shown in Table 12. The regression coefficient of managerial ability in Model 2 of Table 12 is significantly positive (coef. = 0.2293, p<0.01) when the explanatory variable is the managerial shareholding variable (MI). The results indicate that when managers are more competent, they will increase their shareholding levels. Furthermore, the regression coefficient of managerial ability in Model 4 is lower than the coefficient in Model 1 in Table 12. Therefore, the managerial ability is supported by the fact that it influences digital transformation through the equity incentive mechanism, and the equity incentive policy is taken as a mediating effect mechanism.

**Table 12:** The test of mediating effects based on equity incentive mechanism.

	Model 1	Model 2	Model 3	Model 4
	DT	MI	DT	DT
*MA*	0.1273**	0.2293***		0.1243**
	(2.9999)	(6.1710)		(2.8315)
*MI*			0.0389***	0.0384***
			(4.3002)	(4.2215)
*Size*	0.1748***	0.1028***	0.1770***	0.1784***
	(5.2785)	(7.7112)	(5.2781)	(5.3690)
*Age*	-0.2387***	-1.4163***	-0.2188***	-0.2248***
	(-4.1089)	(-20.9145)	(-3.9383)	(-3.8866)
*Board*	0.2079***	0.1418***	0.1988***	0.1990***
	(5.2047)	(4.1971)	(4.7041)	(4.6927)
*Inde*	-0.1445**	-0.5096***	-0.1529**	-0.1535**
	(-2.3698)	(-3.4159)	(-2.3096)	(-2.3515)
*InsInv*	0.0634	-1.4444***	0.1264*	0.1177
	(1.1004)	(-6.7217)	(1.9695)	(1.8248)
*ShBa*	0.0245**	0.2240***	0.0067	0.0066
	(2.6092)	(4.6224)	(0.7957)	(0.8009)
*Dualiy*	0.0266**	0.0634***	0.0215	0.0214
	(2.2653)	(4.7146)	(1.6657)	(1.6372)
*Growth*	0.0649***	0.0926***	0.0641***	0.0607***
	(3.8449)	(3.7446)	(4.1043)	(3.6545)
*Cash*	0.1930	0.0893	0.2072*	0.1789
	(1.7686)	(1.7584)	(1.9275)	(1.5893)
*Constant*	-1.5399**	3.0326***	-1.6835**	-1.7004**
	(-2.4500)	(12.1703)	(-2.5003)	(-2.5243)
*Industry FE*	Yes	Yes	Yes	Yes
*Year FE*	Yes	Yes	Yes	Yes
*N*	14873	14873	14873	14873
*Within-R* ^*2*^	0.4169	0.1289	0.4112	0.4114

Notes:

***Significant at p<0.01,

**Significant at p<0.05,

*Significant at p<0.1.

Based on the analysis of managerial entrepreneurial orientation mechanisms shown in Table 13, the coefficient of managerial ability is significantly positive (coef. = 0.8907, p<0.05) in Model 2. The results indicate that enhanced managerial ability positively contributes to a heightened entrepreneurial orientation among managers. Notably, in Model 4, the coefficient for managerial ability regression remains significantly positive, albeit with a somewhat reduced magnitude of effect compared to Model 1. This observation underscores the influence of managerial ability on digital transformation through the mediation of entrepreneurial orientation mechanisms. In summary, the mediating role of the entrepreneurial orientation mechanism is affirmed, signifying that heightened managerial ability influences the degree of digital transformation within enterprises through the manager’s entrepreneurial orientation.

**Table 13:** The test of mediating effects based on managerial entrepreneurial orientation mechanism.

	Model 1	Model 2	Model 3	Model 4
	DT	EO	DT	DT
*MA*	0.1273**	0.8907**		0.1230**
	(2.9999)	(2.5306)		(2.8602)
*EO*			0.0083***	0.0081***
			(5.1608)	(4.9802)
*Size*	0.1748***	-0.4575***	0.1896***	0.1908***
	(5.2785)	(-6.6150)	(5.3614)	(5.4429)
*Age*	-0.2387***	-3.5821***	-0.2403***	-0.2464***
	(-4.1089)	(-10.3081)	(-4.1010)	(-4.0241)
*Board*	0.2079***	-0.0219	0.2098***	0.2098***
	(5.2047)	(-0.0956)	(4.9636)	(4.9457)
*Inde*	-0.1445**	-0.9244*	-0.1603**	-0.1609**
	(-2.3698)	(-1.9277)	(-2.5776)	(-2.6221)
*InsInv*	0.0634	0.9513***	0.0455	0.0380
	(1.1004)	(5.2276)	(0.7321)	(0.6128)
*ShBa*	0.0245**	0.3571***	0.0124	0.0123
	(2.6092)	(4.0592)	(1.2164)	(1.2227)
*Dualiy*	0.0266**	0.0346	0.0220	0.0219
	(2.2653)	(0.4957)	(1.7531)	(1.7224)
*Growth*	0.0649***	0.0321	0.0664***	0.0629***
	(3.8449)	(0.2658)	(4.6059)	(4.1440)
*Cash*	0.1930	1.5791***	0.2100*	0.1821
	(1.7686)	(6.1463)	(1.9908)	(1.6462)
*Constant*	-1.5399**	20.6626***	-1.8405**	-1.8524**
	(-2.4500)	(10.1743)	(-2.6525)	(-2.6698)
*Industry FE*	Yes	Yes	Yes	Yes
*Year FE*	Yes	Yes	Yes	Yes
*N*	14873	14873	14873	14873
*Within-R* ^*2*^	0.4169	0.0774	0.4109	0.4111

Notes:

***Significant at p<0.01,

**Significant at p<0.05,

*Significant at p<0.1.

## 6. Conclusion

This study employs both internal and external perspectives to scrutinize the influence of managerial ability on digital transformation. Our analysis utilizes a sample of publicly listed companies spanning the years 2009 to 2019. Drawing upon the upper echelon theory and behavioral theory of the firm, we conduct empirical analyses to evaluate the influence of managerial ability on digital transformation. Additionally, we explore the moderating effects of historical aspiration shortfall and industrial competitiveness on the aforementioned relationship, considering both internal and external perspectives. Furthermore, we investigate potential variations across different property rights and corporate governance levels.

This study establishes the following findings: Firstly, managerial ability exerts a positive influence on digital transformation. Secondly, this positive effect is magnified when enterprises confront internal operational challenges, such as historical aspiration shortfalls. However, when enterprises operate within intensely competitive industry environments, the aforementioned positive effects tend to diminish. Thirdly, the positive impact of managerial ability is more pronounced within state-owned enterprises and those characterized by stronger governance level. Lastly, managerial ability enhances digital transformation through two distinct mechanisms: equity incentive and entrepreneurial orientation.

These findings substantiate the varying influence of managerial ability, expand the analytical framework concerning how managerial ability impacts corporate strategy, complement existing research on the precursors of digital transformation, and provide valuable insights into effective management practices within challenging business environments.

### 6.1 Theoretical implications

This paper makes contributions to the extant literature by exploring the impact of management characteristics on corporate strategy from both internal and external vantage points. It validates the pivotal role of managerial ability in driving digital transformation and introduces novel empirical evidence regarding the factors influencing corporate digital transformation.

Firstly, this study fills a gap in the existing literature by delving into the theoretical implications of management characteristics and provides novel empirical insights into digital transformation. Prior research has predominantly examined the impact of explicit characteristics, such as managerial compensation, education, tenure, and career experience, on digital transformation [2, 71]. However, the role of managerial ability, considered as an implicit characteristic, has been relatively understudied. This study innovatively explores the relationship between managerial ability and enterprise digital transformation. By investigating how managerial ability influences digital transformation, it enhances our understanding of the connection between managerial characteristics and enterprise strategy. This suggests that managerial ability shapes long-term innovation decisions, and plays a crucial role in facilitating innovative development within enterprises. Consequently, this study contributes theoretically to the idea that digital transformation, as an innovative behavior exhibited by firms, fundamentally relies on the effective exercise of managerial ability and represents a strategic endeavor aligned with competency tendencies.

Secondly, drawing upon the behavioral theory of the firm, this study augments our comprehension of the impacts exerted by internal operational pressure and external industrial pressure on the execution of organizational innovation strategies. Empirical evidence regarding the role of performance shortfalls in digital transformation has been limited [58]. To comprehend how internal corporate governance can mitigate opportunistic behavior stemming from managers’ short-term orientations, this paper examines the moderating influence of the firm’s internal operational pressure (i.e., historical aspiration shortfalls) on the relationship between managerial ability and digital transformation. Furthermore, recognizing that firm behavior can be influenced by the external business environment, this study explores whether the trait of managerial ability can assume a more effective role in facilitating digital transformation in the context of heightened industrial competitiveness. The historical aspiration shortfall employs a historical approach to establish a reference point, enabling a comparison with past performance, thereby elucidating the impact of internal pressure on managerial ability [72]. In parallel, industrial competitiveness denotes the intensity of competition within the industry, signifying that external environmental pressures can exert an influence on the strategic outcomes of managerial ability. These insights provide a novel perspective on how firms can harness managerial ability to adapt their digital transformation efforts in reaction to changing industrial competitiveness, thereby influencing the innovation decisions of enterprises. As suggested by Ainuaimi et al. [73], we investigate the strategic management actions of managers in implementing digital transformation initiatives, and extend this literature by exploring the boundary conditions of how managerial abilities influence digital transformation. Drawing on the entrepreneurship perspective, this paper reveals the generative effect of managers with high digital transformation capability, which offers novel insights for theoretical development and provides a conceptual foundation for how to achieve digital transformation.

Lastly, we add value to the literature on upper echelons theory and digital transformation research. This study applies managerial ability to the domain of digital transformation research based on the lens of upper echelons theory. Contribution to the behavioral theory of the firm arises from our focus on the joint implications of historical aspiration shortfall and industrial competitiveness for organizational innovation strategies, which previous research has increasingly shown interest in [74]. This research reveals that external developmental challenges exert a more pronounced inhibitory effect on managerial performance compared to internal developmental dilemmas. This underscores the significance of external industry environments. Moreover, our study extends the theoretical framework of the behavioral theory of the firm by illustrating how managerial ability enhances managers’ inclination to engage in digital transformation when firms encounter performance shortfalls. However, this positive effect of managerial ability on digital transformation is attenuated in more competitive external industry contexts. This research augments the theoretical interaction between the upper echelon theory and the behavioral theory of the firm by examining the influence of managerial ability on digital transformation from the dual perspectives of internal and external pressures.

### 6.2 Managerial implications

This study offers some managerial implications for practice. Firstly, in the face of the dynamic and turbulent external market environment, firms need to pay more attention to the managerial ability of managers, recognizing the positive role of managers with a solid capacity to cope with risks, overcome challenges and achieve growth [75]. Managers should develop their abilities according to firm performance and strategic requirements, designing incentive policies and regulating the system accordingly.

Secondly, firms need to provide the foundation for strategic development by adjusting or reallocating organizational resources. Managers should accurately grasp the environmental changes and timely adapt strategies to enhance the dynamic capability to respond to the environment [76]. Meanwhile, managers should possess a risk-aware mindset, enabling them to proactively calibrate the company’s strategic decisions in response to the evolving competitive landscape within the market and the industry. This proactive approach ensures that external environmental pressures do not impede the innovative development of enterprises.

Thirdly, firms should support and lead innovative activities and focus on new technologies and markets. By improving firms’ entrepreneurial orientation, the strategic direction can be altered, and the motivation for organizational change can be increased. Fourthly, managers’ support for digital transformation should be reinforced. According to the environmental shifts and developmental needs, firms should focus on reconfiguring organizational resources and strategic paths to sustain the firm’s growth. In conclusion, in the face of the constantly changing variations in business conditions, managers should have strong resource orchestration ability to facilitate the smooth execution of the digital transformation strategy, help guide firms to establish long-term strategic positioning, and provide better decisions for firms to undertake digital transformation.

### 6.3 Limitations and future research

Several limitations should be mentioned regarding the study. We assessed managerial ability according to the approach proposed by Demerjian et al. [14]. In the future, assessing managerial ability can be approached through various methods, including the amalgamation of case studies and questionnaires, aimed at exploring the diverse dimensions of managerial abilities and discerning the variations in their impacts [77–79]. This inquiry was confined to the sample of Chinese-listed firms. To enhance the generalizability of findings, further studies based on a multicultural context are needed [80]. Subsequent research employing diverse methodologies such as simulations or experiments will contribute to a more profound comprehension of the fundamental mechanisms underpinning these discoveries.

## Supporting information

S1 File (PDF)
